# Risks of Death and Graft Failure After Surgical Versus Percutaneous Coronary Revascularization in Renal Transplant Patients

**DOI:** 10.1161/JAHA.112.003558

**Published:** 2013-02-22

**Authors:** David M. Charytan, Shuling Li, Jiannong Liu, Yang Qiu, Charles A. Herzog

**Affiliations:** 1 Department of Medicine, Brigham and Women's Hospital, Boston, MA (D.M.C.); 2 Cardiovascular Special Studies Center, United States Renal Data System, Minneapolis Medical Research Foundation, Minneapolis, MN (S.L., J.L., Y.Q., C.A.H.); 3 Division of Cardiology, Department of Medicine, Hennepin County Medical Center and University of Minnesota, Minneapolis, MN (C.A.H.)

**Keywords:** coronary disease, kidney, revascularization, surgery, transplantation

## Abstract

**Background:**

Reliable data regarding absolute and relative risks of death and graft failure after coronary artery bypass graft surgery (CABG) and percutaneous coronary intervention (PCI) in renal transplant patients are unavailable.

**Methods and Results:**

Renal transplant patients undergoing inpatient CABG (n=1400) or PCI (n=4097) were identified from United States Renal Data System data. Cumulative incidence of nonfatal graft failure and death was reported for observed events. A Cox model with the Fine–Gray method was used to account for competing risks in assessing relative hazards. Age and race were similarly distributed; patients who underwent CABG were more likely to have acute arrhythmia and congestive heart failure but less likely to have acute myocardial infarction on index admission. In‐hospital death was more frequent after CABG (5.6% versus 3.0%, *P*<0.001). Cumulative incidence of death, graft failure, and the combined outcome at 3 years were 23.1%, 15.4%, and 38.5% after CABG and 22.9%, 13.3%, and 36.1% after PCI, respectively. In adjusted analyses, CABG was not associated with increased risk of graft failure versus PCI during the first 6 months (hazard ratio 1.06, 95% CI 0.79 to 1.43) or from 6 to 36 months (0.98, 0.78 to 1.22). Risk of death increased after CABG during the first 3 months (1.37, 1.08 to 1.73), but decreased from 6 months on (0.76, 0.63 to 0.93).

**Conclusions:**

CABG does not appear to be associated with a difference in risk of graft failure compared with PCI in renal transplant patients. Compared with PCI, adjusted risk of early death is higher after CABG; however, mortality from 6 months on is lower.

## Introduction

Although the risks of cardiovascular events and cardiovascular death are lower in patients with functioning renal allografts than in patients with end‐stage renal disease (ESRD) receiving dialysis,^1^ cardiovascular risks remain high after successful renal transplantation. The cumulative risk of myocardial infarction (MI) is estimated to be as high as 11.1% at 3 years after renal transplant.^2–3^ Other studies suggest that the annual incidence rate of acute coronary syndrome is 6.5 per 1000 patient‐years in patients with functioning renal allografts.^4^ Furthermore, cardiovascular disease remains the leading cause of hospitalization and death in the renal transplant population despite recent medical advances.^1^

These considerations suggest that renal transplant patients may benefit from coronary revascularization, but there are few data to guide the choice of coronary revascularization procedure in this population. Studies in the general population show that coronary artery bypass graft surgery (CABG) provides more complete revascularization and reduces mortality in high‐risk patients compared with percutaneous coronary intervention (PCI), suggesting that CABG should be the preferred revascularization modality.^5^ However, the benefits of CABG relative to PCI in the transplant population could be limited by the dramatically increased incidence of perioperative mortality in patients with abnormal preoperative renal function.^6^ Detrimental effects on renal function may also dramatically alter long‐term outcomes after revascularization. Periprocedural acute kidney injury or periprocedural alteration in the dosing and absorption of immunosuppressive medications may induce graft failure and hasten progression to end‐stage renal disease. Because graft function is strongly correlated with risk of subsequent cardiovascular events,^2,4,7^ a decline in function may subvert the potential for coronary revascularization to reduce long‐term risks. Last, overall quality of life is likely to be negatively affected when patients lose graft function and become dialysis dependent, regardless of whether revascularization is successful in reducing the risk of subsequent MI.

PCI is a minimally invasive alternative to CABG, but whether it is associated with better cardiovascular outcomes or lower risk of graft failure in transplant patients is uncertain. Few studies have compared outcomes of CABG and PCI in the transplant population, and to our knowledge no study has assessed the long‐ and short‐term risks of graft failure after CABG and PCI. Because the choice between CABG and PCI depends on the relative risks of graft failure and death and on the time to each outcome, the current absence of reliable risk estimates in renal transplant patients dramatically limits the ability to make evidence‐based decisions regarding the appropriate revascularization procedure in the transplant setting. This study therefore aimed to compare the long‐ and short‐term risks of death and graft failure in renal transplant patients undergoing CABG or PCI.

## Methods

### Study Population and Follow‐up

Data from the United States Renal Data System (USRDS) were used to identify Medicare fee‐for‐service enrollees (enrolled in Medicare Part A and Part B but not in a health maintenance organization) with renal transplants. Patients undergoing an index revascularization procedure between 2001 and 2009 were included if they underwent first CABG or PCI in an inpatient setting and were Medicare fee‐for‐service enrollees for ≥12 months before the index procedure. During the study period, 4097 PCIs were performed in the inpatient setting and only 395 were performed in the outpatient setting. Thus, nearly all PCIs (91.2%) were performed in the inpatient setting. In combination with other evidence demonstrating that 72% of US interventional cardiologists never performed an outpatient PCI during the first half of study period,^8^ this suggests that outpatient PCIs represent uniquely anatomically and clinically low‐risk patients unlikely to be comparable to patients undergoing CABG. We therefore excluded outpatient PCI procedures from the analysis.

Patients included in the study resided in the United States or its territories. Patients undergoing concomitant valvular surgery or both CABG and PCI at the index procedure were excluded. Patients were followed from the date of the index procedure to the earliest of death, graft failure, subsequent revascularization, 3 years after the index procedure, or December 31, 2010. Dates of death and graft failure were obtained from the USRDS transplant files.

### Medicare Procedures and Comorbid Conditions

*International Classification of Diseases, Ninth Revision, Clinical Modification* (ICD‐9‐CM) and *Current Procedural Terminology* codes on Medicare inpatient claims, outpatient institutional claims, revenue claims, and line items of Part B physician/supplier claims were used to identify CABG, PCI, and procedural characteristics (Table S1). Because all CABGs are performed in the inpatient setting, we excluded patients undergoing PCI as outpatients.

Comorbid conditions were identified from relevant ICD‐9‐CM diagnosis codes on at least 1 Part A inpatient, skilled nursing facility, or home health claim, or on 2 Part A outpatient or Part B claims on different days within the 12 months before the index hospitalization, or on at least 1 Part A inpatient claim during the index hospitalization. For MI, congestive heart failure, and arrhythmia, we distinguished whether the conditions were chronic (previously present before the date of the index admission regardless of acute presentation) or acute (present during the index hospitalization regardless of chronic presentation). Specific types of arrhythmia (ventricular, atrial, and other) and MI (ST elevation, non–ST elevation, and unspecified) were defined for acute presentation using the ICD‐9‐CM diagnosis codes presented in Table S1.

### Statistical Analysis

Baseline demographic and clinical data are presented as counts (percentages). Differences were analyzed using the χ^2^ test. Because death and graft failure were considered competing risks in this study, the cumulative incidence method was used to report the observed event probabilities of each. Visual inspection revealed that the proportionality of graft failure and death risks between CABG and PCI was questionable, and a piecewise Cox regression model was used with the Fine–Gray method^9^ to handle competing risk and adjust for all demographic and comorbid conditions listed in Table 1. The cutoffs of follow‐up intervals were ≤3 months, >3 to ≤6 months, and >6 to 36 months for death and combined death/graft failure and ≤6 months and >6 to 36 months for nonfatal graft failure. Sensitivity analyses were performed to better understand the relative benefits of CABG compared with PCI in important subgroups: (1) comparison of outcomes with off‐pump CABG and PCI; (2) comparison of CABG using an internal mammary artery graft and PCI; (3) comparison of CABG and PCI using a stent; (4) comparison of CABG and PCI using a drug‐eluting stent; and (5) comparison of multivessel CABG and multivessel PCI. Descriptive analyses were performed using SAS version 9.1 (SAS Institute, Cary, NC). The Fine–Gray Cox model was implemented in R package “cmprsk” version 2.15.1 (The R foundation for Statistical Computing). *P*<0.05 was considered significant.

**Table 1.** Baseline Patient Characteristics

Characteristic	CABG (n=1400)	PCI (n=4097)	*P* value
Age, y			0.23
<45	153 (10.9)	522 (12.7)	
45 to 64	737 (52.6)	2122 (51.8)	
65 to 74	439 (31.4)	1222 (29.8)	
≥75	71 (5.1)	231 (5.6)	
Sex			0.02
Men	966 (69.0)	2629 (65.7)	
Race			0.07
White	1158 (82.7)	3305 (80.7)	
Black	178 (12.7)	622 (15.2)	
Other	64 (4.6)	170 (4.2)	
Year			<0.001
2001	230 (16.4)	506 (12.4)	
2002	184 (13.1)	529 (12.9)	
2003	187 (13.4)	471 (11.5)	
2004	153 (10.9)	556 (13.6)	
2005	168 (12.0)	448 (10.9)	
2006	138 (9.9)	469 (11.5)	
2007	107 (7.6)	371 (9.1)	
2008	127 (9.1)	399 (9.7)	
2009	106 (7.6)	348 (8.5)	
Years since transplant			<0.001
<1	110 (7.9)	360 (8.8)	
1 to <3	195 (13.9)	765 (18.7)	
3 to <5	209 (14.9)	766 (18.7)	
5 to <10	492 (35.1)	1255 (30.6)	
≥10	394 (28.1)	951 (23.2)	
Days from admission to surgery			<0.001
0	449 (32.1)	1834 (44.8)	
1	186 (13.3)	805 (19.7)	
2 to 3	279 (19.9)	677 (16.5)	
4 to 6	239 (17.1)	430 (10.5)	
≥7	247 (17.6)	351 (8.6)	
Comorbid conditions			
Acute CHF	356 (25.4)	858 (20.9)	<0.001
Chronic CHF	355 (25.4)	995 (24.3)	0.42
Acute arrhythmia			<0.001
Ventricular	78 (5.6)	199 (4.9)	
Atrial	286 (20.4)	448 (10.9)	
Other	36 (2.6)	146 (3.6)	
Chronic arrhythmia	295 (21.1)	824 (20.1)	0.44
Acute MI			<0.001
STEMI	88 (6.3)	627 (15.3)	
Non‐STEMI	227 (16.2)	763 (18.6)	
Unspecified	20 (1.4)	60 (1.5)	
Chronic MI	324 (23.1)	629 (15.4)	<0.001
ASHD, other than MI	1227 (87.6)	3548 (86.6)	0.32
Other cardiac disease	512 (36.6)	1227 (30.0)	<0.001
CVA/TIA	195 (13.9)	430 (10.5)	<0.001
PVD	436 (31.1)	1267 (30.9)	0.88
Cancer	86 (6.1)	275 (6.7)	0.46
COPD	200 (14.3)	555 (13.6)	0.49
Diabetes	960 (68.6)	2642 (64.5)	0.006
GI disease	103 (7.4)	258 (6.3)	0.17
Liver disease	59 (4.2)	202 (4.9)	0.28

Values are n (%) unless otherwise indicated. Ventricular arrhythmia includes cardiac arrest and coded ventricular arrhythmias. Other arrhythmia includes conduction disorders, unspecified, and cardiac devices. CABG indicates coronary artery bypass graft; PCI, percutaneous coronary intervention; CHF, congestive heart failure; MI, myocardial infarction; STEMI, ST elevation MI; ASHD, atherosclerotic heart disease; CVA/TIA, cerebrovascular accident/transient ischemic attack; PVD, peripheral vascular disease; COPD, chronic obstructive pulmonary disease; GI, gastrointestinal.

Research conducted by the USRDS is classified as exempt under institutional review board regulations.

## Results

### Baseline Characteristics

During the study period (2001–2009), 5497 renal transplant patients underwent a first revascularization. Of these, 1400 (25.5%) underwent CABG and 4097 (74.5%) underwent inpatient PCI. Age and race were similarly distributed between CABG and PCI patients (Table 1), but male sex, diabetes, and stroke were more prevalent in CABG patients. Acute congestive heart failure was present in 25.4% of CABG patients and 20.9% of PCI patients (*P*<0.001); both ventricular (5.6% versus 4.9%) and atrial (20.4% versus 10.9%) arrhythmias were more common in CABG than in PCI patients (*P*<0.001). There were no significant differences in the prevalence of chronic arrhythmia or congestive heart failure. Previously present (chronic) MI was more prevalent in CABG than in PCI patients (23.1% versus 15.4%, *P*<0.001). However, acute MI was more frequent among PCI patients (*P*<0.001) with larger differences in the distribution of ST elevation MI (6.3% versus 15.3%) than in non–ST elevation MI (16.2% versus 18.6%).

### Procedural Characteristics

Most PCI patients received one or more stents; only 402 (9.8%) underwent angioplasty alone (Table 2). Of 3695 patients receiving stents, 1876 (50.8%) received drug‐eluting stents and 1819 (49.2%) received bare metal stents. Most PCIs (3223, 78.7%) involved a single vessel; 541 (38.6%) CABG patients received ≥4 bypass grafts. CABG with internal mammary artery graft was used in most (84.4%) surgeries, and 210 (17.8%) of these procedures were performed off‐pump. Conversely, only 27 (12.4%) of 218 noninternal mammary artery graft procedures were performed off‐pump. Although only a minority of patients were admitted with acute MI, consistent with standard utilization requirements mandating performance of elective procedures within 24 hours of admission under usual circumstances, PCI (64.5%) and CABG (45.3%) were frequently performed within 1 day of admission.

**Table 2.** Procedural Characteristics

Characteristic	n	%
PCI (n=4097)		
No. of arteries		
1	3223	78.7
≥2	874	21.3
No. of stents		
0	402	9.8
1	3213	78.4
≥2	482	11.8
Type of stent		
Bare metal	1819	44.6
Drug eluting	1876	45.8
CABG (n**=**1400)		
No. of vessels		
1	69	4.9
2	263	18.8
3	527	37.6
≥4	541	38.6
Off‐pump	237	16.9
Internal mammary artery graft	1182	84.4

PCI indicates percutaneous coronary intervention; CABG, coronary artery bypass graft.

### Outcomes

**Figure 1.**
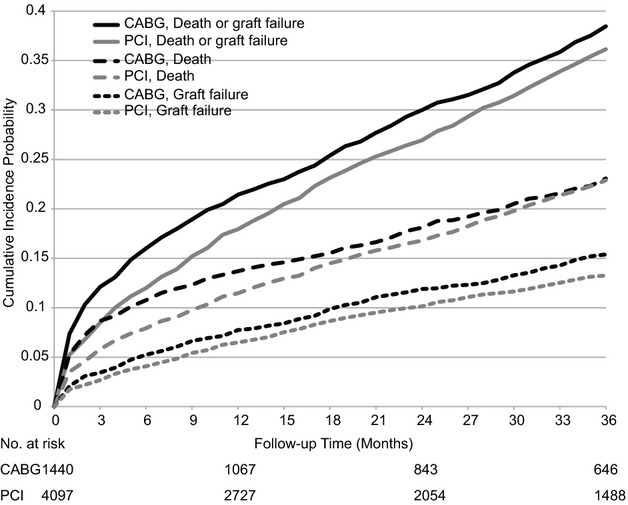
Cumulative incidence of death, nonfatal graft failure, and death or nonfatal graft failure after accounting for competing risks. CABG indicates coronary artery bypass graft; PCI, percutaneous coronary intervention.

More patients died in‐hospital after CABG than after PCI (5.6% versus 3.0%, *P*<0.001). In contrast, overall the 3‐year mortality rate was marginally and nonsignificantly higher after CABG than after PCI (23.1% versus 22.9%, *P*=0.89). Nonfatal graft failure rates differed early during follow‐up but were not significantly higher by 3 years with CABG (15.4%) compared with PCI (13.3%, *P*=0.07). Results for a combined outcome of death or graft failure favored PCI (38.5% versus 36.1%, *P*=0.15; Table 3, Figure 1), but the difference was not significant.

**Table 3.** Cumulative Incidence of Death, Nonfatal Graft Failure, or Combined Death and Graft Failure After Accounting for Competing Risks

Month	Outcome					
	Death		Nonfatal Graft Failure		Death or Graft Failure	
	CABG	PCI	CABG	PCI	CABG	PCI
0	0.000	0.000	0.000	0.000	0.000	0.000
3	0.087	0.058	0.034	0.027	0.121	0.085
6	0.108	0.079	0.053	0.041	0.160	0.120
12	0.137	0.115	0.077	0.065	0.214	0.180
24	0.181	0.168	0.119	0.101	0.300	0.269
36	0.231	0.229	0.154	0.133	0.385	0.361

CABG indicates coronary artery bypass graft; PCI, percutaneous coronary intervention.

After adjustment for comorbid conditions, mortality was significantly higher with CABG from 0 to 3 months (hazard ratio [HR] 1.37, 95% CI 1.08 to 1.73) and significantly lower from 6 to 36 months (HR 0.76, 95% CI 0.63 to 0.93). Risks of nonfatal graft failure were not significantly different with CABG or PCI at 0 to 6 months (HR 1.06, 95% CI 0.79 to 1.43) or from 6 months on (HR 0.98, 95% CI 0.78 to 1.22). Results for the combined outcome of death or graft failure were qualitatively similar to results for death (Table 4).

**Table 4.** Crude and Adjusted Associations of Revascularization Strategy With Outcomes During Follow‐up From a Competing Risk Model

	Death		Nonfatal Graft Failure		Death or Graft Failure	
	HR (95% CI)	*P* value	HR (95% CI)	*P* value	HR (95% CI)	*P* value
Crude (CABG vs PCI), mo						
≤3*	1.51 (1.21 to 1.88)	<0.001	1.29 (0.98 to 1.71)	0.07	1.45 (1.20 to 1.74)	<0.001
3 to ≤6	1.03 (0.67 to 1.58)	0.89	NA	NA	1.16 (0.84 to 1.59)	0.37
>6 to 36†	0.85 (0.71 to 1.02)	0.08	1.15 (0.94 to 1.42)	0.18	0.96 (0.84 to 1.10)	0.56
Adjusted (CABG vs PCI), mo						
≤3*	1.37 (1.08 to 1.73)	0.001	1.06 (0.79 to 1.43)	0.69	1.27 (1.04 to 1.54)	0.018
>3 to ≤6	0.87 (0.56 to 1.35)	0.54	NA	NA	0.95 (0.69 to 1.33)	0.78
>6 to 36†	0.76 (0.63 to 0.93)	0.007	0.98 (0.78 to 1.22)	0.83	0.82 (0.71 to 0.95)	0.007

* Results using cutoffs of ≤3, >3 to ≤6, and >6 to 36 mo (death, combined death/graft failure) and intervals of ≤6 mo *and >6 to 36 mo

† (nonfatal graft failure). Covariates in the model are listed in Table 1. HR indicates hazard ratio; CABG, coronary artery bypass graft; PCI, percutaneous coronary intervention.

Outcomes were qualitatively unchanged in most of the specified subgroups (Table 5), with a few exceptions. Mortality after off‐pump CABG compared with PCI was qualitatively similar to overall risks (HR_6‐36m_ 0.82, 95% CI 0.55 to 1.24), but there was a trend toward a lower risk of nonfatal graft failure (HR_6‐36m_ 0.78, 95% CI 0.48 to 1.27). These findings were not significant, and the number of off‐pump surgeries was small (237). CABG performed with use of an internal mammary artery graft did not alter the risk of graft failure but did appear to confer an even greater survival advantage compared with PCI after 6 months (HR 0.71, 95% CI 0.58 to 0.88).

**Table 5.** Results of Adjusted Sensitivity Analyses

	Death		Nonfatal Graft Failure		Death or Nonfatal Graft Failure	
	HR (95% CI)	*P* value	HR (95% CI)	*P* value	HR (95% CI)	*P* value
Off‐pump CABG vs PCI, mo						
≤3*	1.87 (1.20 to 2.91)	0.006	0.76 (0.39 to 1.52)	0.44	1.32 (0.88 to 1.98)	0.17
>3 to ≤6	0.98 (0.39 to 2.43)	0.96	NA	NA	1.11 (0.57 to 2.14)	0.76
>6 to ≤36	0.82 (0.55 to 1.24)	0.35	0.78 (0.48 to 1.27)	0.31	0.77 (0.57 to 1.05)	0.10
IMG+CABG vs PCI, mo						
≤3*	1.26 (0.97 to 1.63)	0.08	1.06 (0.77 to 1.45)	0.73	1.19 (0.96 to 1.47)	0.11
>3 to ≤6	0.80 (0.49 to 1.31)	0.38	NA	NA	0.91 (0.64 to 1.31)	0.62
>6 to ≤36	0.71 (0.58 to 0.88)	0.002	0.97 (0.77 to 1.23)	0.82	0.79 (0.67 to 0.92)	0.002
CABG vs PCI with stent, mo						
≤3*	1.61 (1.26 to 2.07)	<0.001	1.01 (0.75 to 1.38)	0.93	1.38 (1.13 to 1.70)	0.002
>3 to ≤6	1.00 (0.63 to 1.57)	0.99	NA	NA	1.04 (0.74 to 1.47)	0.82
>6 to ≤36	0.79 (0.64 to 0.96)	0.02	0.94 (0.75 to 1.19)	0.61	0.82 (0.71 to 0.95)	0.008
CABG vs PCI with DES, mo						
≤3*	1.79 (1.27 to 2.51)	<0.001	1.09 (0.72 to 1.63)	0.69	1.61 (1.22 to 2.12)	<0.001
>3 to ≤6	0.87 (0.48 to 1.59)	0.66	NA	NA	0.87 (0.55 to 1.36)	0.54
>6 to ≤36	0.79 (0.60 to 1.02)	0.07	0.96 (0.71 to 1.31)	0.81	0.83 (0.68 to 1.01)	0.06
≥2‐vessel CABG vs ≥2‐vessel PCI, mo						
≤3*	1.43 (0.99 to 2.07)	0.06	1.03 (0.65 to 1.62)	0.91	1.28 (0.95 to 1.72)	0.11
>3 to ≤6	0.88 (0.47 to 1.62)	0.67	NA	NA	0.94 (0.58 to 1.54)	0.82
>6 to ≤36	0.94 (0.69 to 1.29)	0.70	0.86 (0.61 to 1.21)	0.38	0.88 (0.70 to 1.10)	0.25

* Results using cutoffs of ≤3, >3 to ≤6, and >6 to ≤36 mo (death, combined death/graft failure) and intervals of ≤6 mo* and >6 to ≤36 mo (graft failure). HR, hazard ratio; CABG, coronary artery bypass graft; PCI, percutaneous coronary intervention; IMG, internal mammary artery graft; DES, drug‐eluting stent; PTCA, percutaneous transluminal coronary angioplasty (without stent); STEMI, ST elevation myocardial infarction.

Finally, we compared results for 874 PCI and 1331 CABG patients undergoing revascularization of ≥2 vessels. Although graft outcomes were unchanged within this subgroup, there was a suggestion of an attenuation of the mortality benefit from CABG at ≥6 months (HR 0.94, 95% CI 0.69 to 1.29).

## Discussion

An optimal coronary revascularization strategy for patients with functioning renal allografts should balance both cardiovascular and renal outcomes. However, data on overall and graft survival in renal transplant patients undergoing PCI and CABG are currently insufficient for reliable assessment of the relative risks of these critical clinical outcomes. We therefore analyzed postrevascularization outcomes in a large contemporary cohort of patients with functioning renal allografts undergoing a first coronary revascularization procedure.

Although overall survival at 3 years was 0.2% lower after CABG than after PCI and crude risks of death, nonfatal graft failure, and the combined outcome of death and graft failure did not suggest significant benefits from CABG compared with PCI, the lack of benefit appeared to be largely explained by the underlying demographic and clinical characteristics of the PCI and CABG groups. After full adjustment, we found no significant differences in the risk of nonfatal graft failure, increased early mortality, and decreased risk of death or the combined outcome beyond 6 months after CABG compared with PCI. Thus, our results are consistent with an increase in periprocedural risks with CABG but with an accumulating, protective benefit of surgical compared with PCI.

Multiple subgroup analyses were conducted to better understand the robustness and determinants of these findings. We examined subgroups of patients undergoing multivessel PCI, off‐pump CABG, PCI with insertion of a drug‐eluting stent, PCI with insertion on any stent, and multivessel revascularization. Results within these subgroups were generally consistent with our overall findings, and they provided some intriguing findings. Our analyses suggest a possible protective effect of off‐pump surgery on graft function. This finding requires confirmation in larger cohorts. However, it is consistent with recent studies showing decreased risks of acute kidney injury after off‐pump surgery in the general population^10^ and suggests the hypothesis that off‐pump surgery may be preferable in renal transplant patients. The survival benefit of CABG compared with PCI was increased slightly for patients who received an internal mammary artery graft during CABG, but the advantages of CABG were minimized compared with multivessel stenting. Both findings are consistent with studies in the general population demonstrating improved survival with the use of arterial grafts^11^ or more complete revascularization.^12–13^ These findings require confirmation in larger, preferably randomized cohorts, but they provide support for the concept that within the renal transplant population, CABG provides maximal benefits compared with PCI when arterial revascularization of the left anterior descending is feasible, but that PCI may be a good alternative when complete or near complete revascularization can be accomplished percutaneously or when vein grafts are the only feasible options.

Studies conducted in the general population suggest that CABG is a superior treatment to PCI^14–15^; however, it is unlikely that any significant numbers of renal transplant patients participated in those trials, and whether the findings apply to renal transplant patients is uncertain. Graft function is also a well‐established risk factor for cardiovascular events and death.^2,4,16–17^ Thus, a higher likelihood of periprocedural allograft injury after CABG than after PCI could result in the acceleration of graft failure and an increase in the associated, long‐term risks of cardiovascular events. Furthermore, transplant patients may place a premium on maintaining graft function and may prefer procedures with higher long‐term risks of death but lower risk of permanent dialysis dependence.

Despite the clear need for reliable estimates of the relative risks of postrevascularization death and graft failure, we are aware of only a single prior report analyzing the risk of graft failure after coronary revascularization. In that study, there was no difference in the risk of graft failure among 45 renal transplant patients from a single center who underwent CABG compared with PCI between 1968 and 1994.^18^ However, a more recent analysis of a cohort of 57 renal transplant patients undergoing CABG suggested that the risk of graft failure may be substantial after CABG; acute graft failure occurred in 14% of patients, and 40% of the surviving patients with acute graft failure required permanent dialysis.^19^ Our study expands on this literature by demonstrating in a large contemporary cohort that the overall incidence of permanent, nonfatal graft failure after revascularization is low (<20% at 3 years). Our cohort included patients revascularized as recently as 2009, and our findings are likely to be more applicable to contemporary surgical and percutaneous practices than are findings of prior studies. Furthermore, the larger size of our cohort allowed us to adjust for relevant confounders and to determine that there were no significant differences in the adjusted risk of nonfatal graft failure with PCI compared with CABG. Comparative data on overall survival (or death and graft failure) are also sparse. In a previous study, Herzog et al^20^ found no significant difference in the survival of renal transplant patients after PCI compared with CABG but, in agreement with our analysis, found that CABG using internal mammary artery grafts was associated with improved survival compared with PCI. These consistent findings across both studies may be explained by the better durability of arterial conduits,^11^ and they suggest that the use of an internal mammary graft may be the preferred revascularization strategy in transplant patients, particularly those with high‐risk coronary anatomy. In contrast to the current study, the previous study by Herzog et al^20^ found no significant survival advantage from internal mammary graft–negative CABG, but the point estimate was consistent with a 20% improvement in mortality. This finding is similar to the 24% risk reduction observed in the current analysis. We thus believe that both studies provide support for a benefit from CABG compared with PCI, with the divergent statistical findings most likely explained by the increased power provided by the greater number of CABG and PCI patients included in the current study.

Randomized trials comparing CABG and PCI are needed for further clarification of these issues. Unfortunately, it is unlikely that trials in the transplant population will be conducted in the foreseeable future. In their absence, our analysis may guide clinicians and patients deciding between procedures and should provide reassurance that despite an increase in short‐term risks, long‐term survival is likely to be better and renal outcomes to be equivalent after CABG compared with PCI. Furthermore, although they require confirmation, our sensitivity analyses provide preliminary evidence that the relative advantages of CABG may be even greater when patients are able to undergo off‐pump surgery or are candidates for internal mammary grafting.

An important strength of our study is the large sample size; to our knowledge, we assembled by far the largest cohort of revascularized renal transplant patients studied to date. This study sample was also more contemporary and nationally representative than those analyzed in previous investigations. This large sample size allowed for extensive comorbidity adjustment and an exploration of outcomes within numerous important subgroups.

Our study is also limited in important ways. First, we excluded patients undergoing outpatient PCI or CABG; extrapolation of our findings to revascularization performed in the outpatient setting may not be appropriate. Our multivariable models were designed to correct for imbalances in baseline patient characteristics, but they may have been unable to fully correct for biases in the selection and referral of patients for CABG versus PCI or for unmeasured confounders accounting for differences in post‐CABG and post‐PCI outcomes. The absence of detailed clinical data such as functional status, ejection fraction, or results of coronary angiography and stress tests additionally limits our ability to account for important factors that could influence the choice of procedure or outcome. Of note, although we were able to adjust for number of vessels revascularized, we were unable to adjust for completeness of revascularization. Data on baseline glomerular filtration rate would also have been particularly useful for assessing the risks of graft failure but were unavailable. Finally, comorbid conditions and outcomes were assessed from administrative data and not directly from clinical records. However, previous studies have demonstrated strong associations with outcomes^21^ and high specificity for the identification of various medical conditions when diagnostic codes are used to define comorbid conditions.^22–23^

The use of administrative data also limited outcomes assessment. Although we were able to examine graft failure, we were unable to identify other important renal outcomes, such as a 50% rise in serum creatinine progression across chronic kidney disease. Finally, the number of patients receiving multivessel PCI was small, thereby limiting our ability to make conclusions about the relative merits of CABG compared with multivessel PCI. Additional randomized trials and studies using clinically richer databases to assess baseline characteristics are warranted to confirm our findings and identify potential effect modifiers.

In conclusion, we compared outcomes of CABG and PCI in patients with renal transplants. We found that nonfatal graft failure affected <20% of patients during the first 3 years of follow‐up and occurred less frequently than death. Although early risks of death were higher with CABG, mortality after 6 months and the combined outcome were significantly lower after CABG than after PCI, and the risk of graft failure was not significantly different between CABG and PCI. These findings suggest that the risk of graft failure should not be an important determinant in choosing coronary revascularization procedures for renal transplant patients and that CABG may be favored when feasible and other comorbid conditions are not otherwise expected to unduly shorten survival. PCI is a reasonable alternative for renal transplant patients for whom the primary outcome of interest is short‐term survival. These findings have important implications for the care of renal transplant patients, and confirmation in additional studies is warranted.
